# Spatio-temporal connectivity of the aquatic microbiome associated with cyanobacterial blooms along a Great Lake riverine-lacustrine continuum

**DOI:** 10.3389/fmicb.2023.1073753

**Published:** 2023-02-09

**Authors:** Sophie Crevecoeur, Thomas A. Edge, Linet Cynthia Watson, Susan B. Watson, Charles W. Greer, Jan J. H. Ciborowski, Ngan Diep, Alice Dove, Kenneth G. Drouillard, Thijs Frenken, Robert Michael McKay, Arthur Zastepa, Jérôme Comte

**Affiliations:** ^1^Watershed Hydrology and Ecology Research Division, Environment and Climate Change Canada, Burlington, ON, Canada; ^2^Department of Biology, McMaster University, Hamilton, ON, Canada; ^3^Department of Biology, Trent University, Peterborough, ON, Canada; ^4^Energy, Mining and Environment, National Research Council of Canada, Montreal, QC, Canada; ^5^Department of Integrative Biology, University of Windsor, Windsor, ON, Canada; ^6^Department of Biological Sciences University of Calgary, Calgary, AB, Canada; ^7^Ontario Ministry of the Environment, Conservation and Parks, Environmental Monitoring and Reporting Branch, Etobicoke, ON, Canada; ^8^Great Lakes Institute for Environmental Research, University of Windsor, Windsor, ON, Canada; ^9^Cluster Nature & Society, HAS University of Applied Sciences, s-Hertogenbosch, Netherlands; ^10^Great Lakes Center for Fresh Waters and Human Health, Bowling Green State University, Bowling Green, OH, United States; ^11^Centre Eau Terre Environnement, Institut National de la Recherche Scientifique, Quebec City, QC, Canada; ^12^Groupe de Recherche Interuniversitaire en Limnologie et en Environnement Aquatique (GRIL), Université de Montréal, Montreal, QC, Canada

**Keywords:** aquatic microbiome, cyanobacteria, Lake Erie watershed, harmful algal blooms, genetic connectivity

## Abstract

Lake Erie is subject to recurring events of cyanobacterial harmful algal blooms (cHABs), but measures of nutrients and total phytoplankton biomass seem to be poor predictors of cHABs when taken individually. A more integrated approach at the watershed scale may improve our understanding of the conditions that lead to bloom formation, such as assessing the physico-chemical and biological factors that influence the lake microbial community, as well as identifying the linkages between Lake Erie and the surrounding watershed. Within the scope of the Government of Canada’s Genomics Research and Development Initiative (GRDI) Ecobiomics project, we used high-throughput sequencing of the 16S rRNA gene to characterize the spatio-temporal variability of the aquatic microbiome in the Thames River–Lake St. Clair-Detroit River–Lake Erie aquatic corridor. We found that the aquatic microbiome was structured along the flow path and influenced mainly by higher nutrient concentrations in the Thames River, and higher temperature and pH downstream in Lake St. Clair and Lake Erie. The same dominant bacterial phyla were detected along the water continuum, changing only in relative abundance. At finer taxonomical level, however, there was a clear shift in the cyanobacterial community, with *Planktothrix* dominating in the Thames River and *Microcystis* and *Synechococcus* in Lake St. Clair and Lake Erie. Mantel correlations highlighted the importance of geographic distance in shaping the microbial community structure. The fact that a high proportion of microbial sequences found in the Western Basin of Lake Erie were also identified in the Thames River, indicated a high degree of connectivity and dispersal within the system, where mass effect induced by passive transport play an important role in microbial community assembly. Nevertheless, some cyanobacterial amplicon sequence variants (ASVs) related to *Microcystis*, representing less than 0.1% of relative abundance in the upstream Thames River, became dominant in Lake St. Clair and Erie, suggesting selection of those ASVs based on the lake conditions. Their extremely low relative abundances in the Thames suggest additional sources are likely to contribute to the rapid development of summer and fall blooms in the Western Basin of Lake Erie. Collectively, these results, which can be applied to other watersheds, improve our understanding of the factors influencing aquatic microbial community assembly and provide new perspectives on how to better understand the occurrence of cHABs in Lake Erie and elsewhere.

## 1. Introduction

Cyanobacterial harmful algal blooms (cHABs) represent a major threat to waterbodies worldwide and are increasing in frequency, magnitude, and duration (Taranu et al., 2015; Huisman et al., 2018; Ho et al., 2019). cHABs are a major concern for lake management because, beside greatly impairing water quality for recreational and fisheries purposes, compromising the safety of drinking water, they can also produce toxins and secondary metabolites (Paerl and Otten, 2013) that may cause fatalities or serious health issues for humans and animals in contact with the contaminated water (Carmichael, 2001; Martínez Hernández et al., 2009; Backer et al., 2013). Therefore, identifying the principal factors that cause and mitigate cHABs is a high priority need for assessing ecosystems current and future health.

Nutrient loads, especially phosphorus (P), have been identified as the main cause of cyanobacterial blooms in most inland waters (Downing et al., 2001; Stumpf et al., 2012; Michalak et al., 2013; Scavia et al., 2014). However, there is increasing evidence that abiotic factors alone do not explain and predict the occurrence of cHABs (Paerl et al., 2016; Woodhouse et al., 2016). Indeed, a decrease in external P loading to impaired waterbodies does not always result in the disappearance of cHABs, and often results in hysteresis with recurrent outbreaks of these blooms (Berthold and Campbell, 2021). Intense eutrophication often results in a regime shift and legacy of nutrient-rich sediments, which can act as an internal source and continue to fuel blooms despite efforts to reduce external loading (Matisoff et al., 2016). Nutrient stoichiometry (e.g., N:P) can also plays a role in bloom size, composition and toxin content, and low N:P ratios in eutrophic lakes have been associated with increased toxin concentration in blooms (Zastepa et al., 2017). While the idea of dual nutrient management is gaining traction (Paerl et al., 2016), a recent provocative claim that P-only management will lead to more toxin production (Hellweger et al., 2022), has been challenged for not taking into account *in situ* lake processes and responses of phytoplankton communities (Huisman et al., 2022; Stow et al., 2022).

Lake Erie has experienced multiple cHAB events during the last few decades and still suffers from human impact despite the implementation of remediation action plans (Watson et al., 2016). Severe eutrophication of Lake Erie dates back to the 1950s, and the lake experienced major lake-wide blooms in the 1960s and 1970s (Allinger and Reavie, 2013). These were dominated by eukaryotic algae (notably diatoms, chlorophytes, and dinoflagellates) together with N-fixing (*Aphanizomenon* and *Dolichospermum*) and non N-fixing cyanobacteria (notably *Planktothrix*, *Microcystis*, and *Pseudanabaena*; Munawar et al., 2008; Allinger and Reavie, 2013). High external point-source inputs of P were identified as the primary cause of these events, and in 1972, a binational effort was established under the Canada-USA Great Lakes Water Quality Agreement (GLWQA), which successfully reduced loading to meet phosphorus reduction targets and resulted in a significant decline in Lake Erie blooms (Scavia et al., 2014). However, since the mid 1990’s, there has been a significant increase in diffuse loading - notably of highly bioavailable forms of P and N. Furthermore, lake-wide colonization by dreissenid mussels has, despite increasing water transparency, radically altered the nearshore-offshore nutrient exchange by excreting and enhancing P input directly around the substrate they colonized (Watson et al., 2016). These factors have been linked with a resurgence of blooms, and shift in dominance toward toxin-producing cHABs – notably, species of non-diazotrophic *Microcystis* and *Planktothrix* (Davis et al., 2014; Harke et al., 2016; Davenport et al., 2019), and diazotrophs such as *Dolichospermum* (Michalak et al., 2013). However, modeling and forecasting still lead to substantial uncertainties related to cHABS extent and variability in Lake Erie (Obenour et al., 2014), which suggests that the functioning of the ecosystem as a whole is not well understood and that an integrated approach is needed to understand how the lake ecosystem will respond to future changes.

The ‘lake as a microcosm’ (Forbes, 1887) is a fundamental concept in limnology, which contributes to our understanding of in-lake biological and physico-chemical interactions. However, this is an overly simplistic representation of lakes (and many other waterbodies), which are in fact integrated within watersheds and are thus influenced by large-scale processes such as climate perturbations (Adrian et al., 2009), hydrology (Martin and Soranno, 2006), and land-use allocation (Walsh et al., 2003). The connection between the lake and watershed processes means that management action on land and/or in tributaries will have repercussions on the downstream lake ecosystem. For example, nutrient loads from Lake Erie’s watershed directly and indirectly impact in-lake nutrient concentrations (Maccoux et al., 2016), that have the potential to support cHABs (Guo et al., 2021). However, the role(s) of microbial communities in mediating drainage inputs - and how they affect downstream events of cHAB - are poorly understood. Therefore, there is a need to assess the spatio-temporal variability of the aquatic microbial communities (i.e., aquatic microbiome) within the Lake Erie watershed in order to better understand the biological and ecological contexts underlying cHAB occurrences. Additionally, the role of the co-occurring microbes is often overlooked when studying cHABs (Mou et al., 2013; Pound et al., 2021), despite their key involvement in carbon and nutrient cycling (Falkowski et al., 2008), and in bloom and toxin degradation (Shao et al., 2014; Dziga et al., 2019; Salter et al., 2021).

In this study, we took an integrated approach and used high-throughput sequencing of the 16S rRNA gene to characterize spatio-temporal variation of the aquatic microbiome in Lake Erie and its connectivity with watershed components from the upstream Huron-Erie corridor, focusing on the Canadian side. We also examined the structure of the cyanobacterial community to gain insight on the factors influencing the development of cHABs. Watershed integration involved sampling the Thames River, Lake St. Clair, Detroit River, and Lake Erie, which altogether represent a more than 500 km long hydrological continuum spanning a strong environmental gradient, reflecting land use. The Thames River transports elevated concentrations of nutrients, draining 6,000 km^2^ of agricultural land in southwestern Ontario (Leach, 1980). Amongst Canadian tributaries to Lake St. Clair and Lake Erie, the Thames has the highest discharge and is the largest contributor of P (Maccoux et al., 2016; Kao et al., 2022). With this framework, we aim to uncover the spatial and environmental factors shaping the microbial community within Lake Erie watershed, and assess the role of selection or dispersion and passive transport of cells from upstream to downstream. We hypothesized that, despite the extended geographic distance and gradient in environmental variables, upstream watershed input can be an important source of recruitment for Lake Erie’s microbial and cyanobacterial communities.

## 2. Methods

### 2.1. Study sites and physicochemical variables

**Figure 1:**
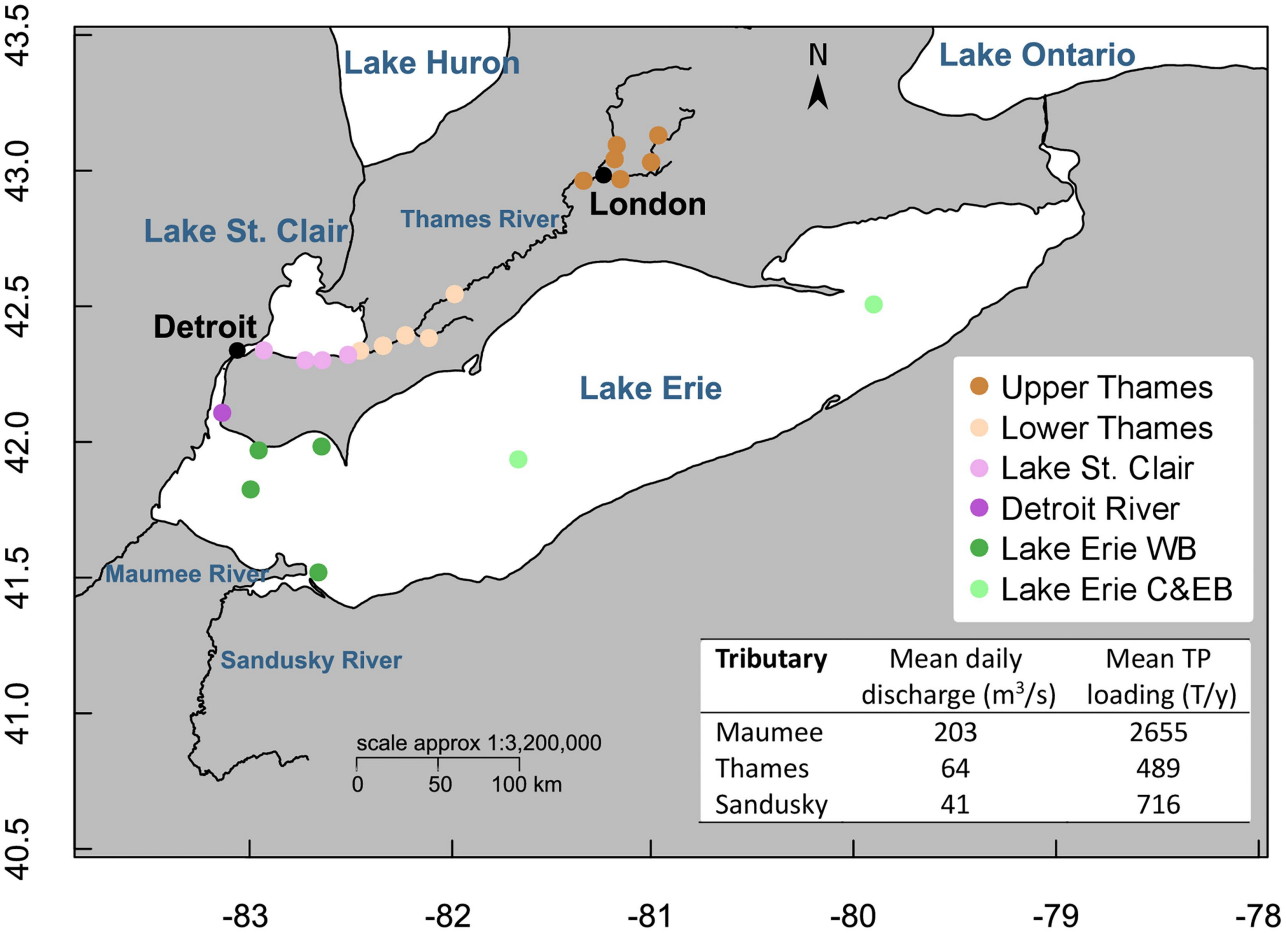
Location of the sampling sites colored by systems (Upper and Lower Thames Rivers, Lake St. Clair, Detroit River and Lake Erie). Map created with R using the open-access databases “worldHires” (https://www.evl.uic.edu/pape/data/WDB/) and river line downloaded from the Government of Canada Open Data Portal (http://open.canada.ca/en/open-data) and the National Weather service (https://www.weather.gov/gis/Rivers). Estimates of mean daily discharge from 2016 to 2019 were calculated from data available from the Water Survey of Canada (https://wateroffice.ec.gc.ca/) for the Thames River and from the U.S. National Water Information System (https://waterdata.usgs.gov/nwis) for the Maumee and Sandusky Rivers. Estimates of mean TP loads from 2016 to 2019 were calculated from the data available from ErieStat (https://www.blueaccounting.org/).

A total of 285 water samples (93 in the Upper Thames River, 47 in the Lower Thames, 70 in Lake St. Clair, 14 in the Detroit River, and 61 in Lake Erie) were collected between January 2016 and October 2019 (sampling frequency detailed in Supplementary Table S1). Samples were taken repeatedly at the same sites over the years and seasons encompassing different systems defined as the Upper and Lower Thames River, Lake St. Clair, Detroit River, and the Western, Central and Eastern basins of Lake Erie (Figure 1). Surface samples were collected in triplicate using a Niskin bottle in a rosette configuration for Lake Erie, a Van Dorn bottle for Lake St. Clair and the Detroit River, and from the shore or from a bridge with a pole holding sterile bottles for the Thames River. All samples were kept in a cooler on ice and filtered within 24 h on board the Canadian Coast Guard Ship (CCGS) *Limnos* for Lake Erie and in the lab for the other systems. Temperature (Temp), specific conductivity (Cond), pH, and dissolved oxygen (DO) were measured at each site with a SBE 25plus Sealogger (Seabird) on board the CCGS Limnos, and with a Quatro Pro (YSI Inc.) in Lake St. Clair, Detroit and Thames Rivers. Water samples for total phosphorus (TP), total dissolved phosphorus (TDP), total nitrogen (TN), total dissolved nitrogen (TDN), and particulate organic carbon (POC), were submitted to Environment and Climate Change Canada’s National Laboratory for Analytical Testing (NLET, Burlington, Ontario) and prepared following their Standard Operating Procedures for filtration and preservation. Chlorophyll a (chl*a*) concentrations were determined spectrophotometrically after extraction in acetone following the NLET method (NLET, 1997; Table 1).

**Table 1:** Average (SD in brackets) of the environmental variables for each ecosystem and across seasons: pH, temperature (Temp), dissolved oxygen (DO), conductivity (Cond), total phosphorus (TP), total dissolved phosphorus (TDP), total nitrogen (TN), total dissolved nitrogen (TDN), chlorophyll a (Chla).

System	Season	pH	Temp (°C)	DO (mgL^−1^)	Chla (μgL^−1^)	Cond (μScm^−1^)	TP (μgL^−1^)	TDP (μgL^−1^)	TN (mgL^−1^)	TDN (mgL^−1^)
Upper Thames	Winter	8.2 (0.2)	2.9 (1.8)	-	1.9 (1.1)	479 (168)	151 (144)	112 (103)	-	9 (1.8)
	Spring	8.2 (0.2)	12.3 (7.3)	-	18.7 (7.8)	593 (115)	93 (38)	51 (30)	7.9 (1)	7.4 (1.7)
	Summer	8.3 (0.3)	21.2 (2.7)	-	11.6 (20.9)	707 (113)	90 (60)	49 (32)	4.9 (2.2)	5.5 (2.5)
	Fall	8.3 (0.3)	15.2 (2.5)	-	100.1 (203)	679 (137)	159 (136)	41 (27)	5.3 (1.2)	4.5 (1.6)
Lower Thames	Winter	8.2 (0.1)	2.5 (1.9)	-	1.1 (0)	576 (86)	188 (99)	88 (26)	-	7.9 (0.8)
	Spring	8.2 (0.3)	15.1 (6.7)	-	48.7 (41.7)	589 (110)	242 (174)	66 (50)	7.4 (0.3)	6.1 (1)
	Summer	8.1 (0.3)	23.3 (2.4)	-	7.6 (7.5)	676 (108)	127 (91)	44 (27)	4.9 (1.2)	4.1 (1.3)
	Fall	8.4 (0.3)	15.9 (3)	-	16.6 (7.8)	696 (61)	103 (36)	32 (17)	3.8 (0.6)	3.4 (0.8)
Lake St. Clair	Winter	⊗	⊗	⊗	⊗	⊗	⊗	⊗	⊗	⊗
	Spring	8.2 (0.3)	19.6 (2.6)	9.9 (−)	15.3 (18.4)	356 (157)	57 (65)	23 (37)	2.7 (2.6)	2.6 (2.5)
	Summer	8.5 (0.4)	23.2 (2.6)	7 (1.1)	10.5 (8.5)	326 (156)	40 (26)	10 (14)	1.3 (1.4)	1.1 (1.3)
	Fall	7.9 (0.5)	17.2 (3.7)	3.8 (0.9)	6 (8.1)	288 (89)	71 (88)	28 (43)	0.7 (0.4)	0.5 (0.3)
Detroit River	Winter	⊗	⊗	⊗	⊗	⊗	⊗	⊗	⊗	⊗
	Spring	8.1 (−)	17.6 (−)	10 (−)	1.4 (−)	194 (−)	11 (−)	2 (−)	0.6 (−)	0.6 (−)
	Summer	8.6 (0.5)	22.3 (2.2)	8.2 (1.5)	1.9 (1.5)	217 (16)	14 (2)	4 (2)	0.6 (0.1)	0.5 (0.1)
	Fall	8.3 (0.2)	18 (0.8)	5.5 (−)	1.5 (0)	201 (2)	18 (12)	6 (5)	0.4 (−)	0.4 (−)
Lake Erie WB	Winter	-	-	-	-	-	-	-	-	-
	Spring	8.2 (0.1)	11.8 (5)	10.4 (1.1)	2.3 (1)	260 (26)	24 (23)	8 (9)	1.3 (0.6)	0.8 (0.7)
	Summer	8.6 (0.3)	23.6 (1.5)	8.1 (0.9)	6.1 (6.1)	231 (26)	18 (9)	6 (9)	0.5 (0.1)	0.4 (0.1)
	Fall	8.4 (0.3)	19.9 (1.1)	8.2 (1.2)	5.2 (3.3)	228 (19)	19 (10)	6 (4)	0.4 (0.1)	0.3 (0.1)
Lake Erie C&EB	Winter	8.2 (−)	-1 (−)	-	1 (−)	215 (−)	15 (−)	10 (−)	0.4 (−)	0.4 (−)
	Spring	8.3 (0.2)	6.7 (1.6)	-	2.9 (3.4)	284 (15)	13 (6)	5 (2)	0.4 (0.1)	0.3 (0.1)
	Summer	8.6 (0.1)	23 (1.4)	-	1.9 (1.7)	272 (5)	7 (2)	3 (0.6)	0.4 (0.1)	0.3 (0.1)
	Fall	8 (0.1)	18.9 (0.6)	-	6.2 (4.3)	274 (5)	19 (13)	6 (4)	0.4 (0)	0.3 (0)

Absence of value is either because the data was not measured for the sample (−) or because the sample was not taken (⊗).

Estimates of mean daily discharge from 2016 to 2019 were calculated from data available from the Water Survey of Canada^1^ for the Thames River and from the U.S. National Water Information System^2^ for the Maumee, Sandusky, St. Clair, and Detroit Rivers.

### 2.2. DNA sampling and amplicon sequencing processing

From 250 to 300 ml of water for DNA extraction was filtered in triplicate through 0.2 μm Polyethersulfone filters (Fisher Scientific) and stored at −80°C until further processing. All genomic procedures were carried out following a standard approach for genomic research conducted by the government of Canada (Edge et al., 2020). DNA was extracted using the Qiagen DNAEasy PowerSoil DNA isolation kits following instructions from the manufacturer. The V4–V5 hyper-variable regions of the 16S rRNA gene were amplified with primers 515F (GTGCCAGCMGCCGCGGTAA) and 926R (CCGYCAATTYMTTTRAGTTT). Libraries were prepared in the Energy, Mining and Environment Biotechnology Research Center of the National Research Council of Canada (Montreal, Quebec). All triplicate samples were sequenced on an Illumina MiSeq platform at the National Research Council (Saskatoon, Saskatchewan). The 16S rRNA gene sequences were analyzed in R Core Team (2018) following the dada2 pipeline (Callahan et al., 2016), using the high-performance computing environment of Shared Services Canada in Dorval, Quebec (Edge et al., 2020). First, non-biological sequences of the primers were removed with cutadapt (Martin, 2012). Then, for each sequencing plate, raw read quality profiles were assessed and the low-quality bases at the end of the read were trimmed with a truncQ score of 11, as suggested for large datasets. Sequences with a maximum expected error (maxEE) greater than 2 were removed and high-quality sequences were merged in an amplicon sequence variant (ASV) table. Chimeras were removed with the ‘removeBimeraDenovo’ command and taxonomy was assigned with the Silva database version 128 released in 2016 (Yilmaz et al., 2014). Additionally, a Barcode of Life Data System (BOLD) reference database that was developed using sequences of cyanobacterial and algal cultures, was used to classify sequences belonging to the Cyanobacteria phylum (Ivanova et al., 2019). Sequences corresponding to Archaea, Chloroplasts, and Eukaryotes were removed from the ASV table and identical sequences that only varied in length were collapsed with the ‘collapseNoMismatch’ command in dada2. The ASV table was rarefied a hundred times at 10,000 reads per sample with the ‘rarefy_even_depth’ command available in the package phyloseq version 1.36.0 (McMurdie and Holmes, 2013), and the average read counts of the 100 tables was used for downstream analyses. This process discarded 383 ASVs out of 68,230 and 50 samples, which when taking into account the number of samples that were collected and sequenced in triplicate, represented a loss of only 9 biological samples.

### 2.3. Statistical analyses

Dispersion plots for visualizing variation across systems, seasons, and years were generated with the ‘betadisper’ function available in the R package vegan version 2.6.2 (Oksanen et al., 2019). Non metric multidimensional scaling (NMDS) was generated using the command ‘metaMDS’ available in the R package vegan (Oksanen et al., 2019) and based on Bray-Curtis distances on the squared-root ASV table. To examine how environmental variables correlated with the community ordination, we selected the environmental variables measured in all systems (i.e., Cond, pH, temperature, TP, TDP, TN, TDN, POC, and chl*a*) based on the following: a matrix of Spearman correlations among all environmental variables was calculated and those with high correlation (ρ > 0.8 and *p* < 0.01) with more than one other variable were removed. Hence, TDP and TN were removed for downstream analyses. The remaining environmental variables were projected onto the NMDS ordination using envfit function available in package vegan (Oksanen et al., 2019). Significant differences in microbial community composition as a function of the system sampled or the season were tested with PERMANOVA (Adonis function in package vegan) with 999 permutations.

Mantel correlations were used to assess correlations between geographic distances and structure of the microbial and cyanobacterial communities. Community matrices were first Hellinger transformed with the decostand command in vegan and then detrended (Borcard et al., 2018). Distances between each sampling site coordinate were calculated with the ‘pointDistance’ function available in R package raster version 3.6.3 (Hijmans, 2020) while accounting for the flow direction (from the most upstream to the most downstream site). Additionally, a variation partitioning analysis was employed to disentangle the proportion of variation observed in the microbial community that was due solely to environmental variables or spatial variability. This analysis was performed using the ‘varpart’ function and by transforming geographic coordinates into principal coordinates of neighbor matrices (PCNM) with the ‘pcnm’ function (both are available in the R package vegan; Oksanen et al., 2019). PCNM allows for the detection of any type of spatial patterns and is not restricted to linear ones (Borcard and Legendre, 2002). For this analysis, only environmental variables that were measured in all systems were used (Cond, pH, temperature, TP, TDP, and TDN), and samples containing missing data were removed (Table 1). All environmental variables and PCNMs were selected with the forward.sel function in the adespatial version 0.3.19 package (Dray et al., 2018) prior to being included in the variation partitioning analysis. Consequently, the analysis was performed on 231 samples (85 from the Upper Thames, 40 from the Lower Thames, 38 from Lake St. Clair, 12 from Detroit River, and 56 from Lake Erie).

Stacked bar plots of the microbial and cyanobacterial community composition, merged as a function of the system to improve plot clarity, were drawn with ggplot2 version 3.3.6 (Wickham, 2016). Sparse Correlations for Compositional data (SparCC) at the phylum level between the relative abundance of cyanobacterial and the most abundant bacterial groups, were produced with the sparcc command in the package SpiecEasi version 1.1.2 (Kurtz et al., 2022). Shared taxa across seasons and systems were represented with and upset plot from the UpSetR package version 1.4.0 (Gehlenborg, 2019).

A phylogenetic analysis of the 15 most abundant cyanobacterial ASVs was performed with sequences from the BOLD database (Ivanova et al., 2019) and from GenBank release 239 (Benson et al., 2018). Two Escherichia coli 16S sequences were used as outgroup (accession number HG917881 and HF584705). Sequences were first aligned with MUSCLE algorithm available in the software mega11. The best molecular model was tested with mega and a consensus neighbor-joining tree was then built, based on 1,000 trees, with associated bootstrap values using the best fit model Kimura 2 with gamma parameter.

Generalized additive models (GAMs) were used to assess the relationship between the proportion (from 0 to 1) of cyanobacterial genera relative abundance (arcsine and squared-root transformed) and the following environmental variables: temperature, pH, log-transformed TP, TDP, TN, TDN, and the ratio between TN and TP. GAMs have the advantage of mixing linear model terms with a smoothed and flexible model term and are therefore best suited for non-linear relationships because they can fit non-linear models to data (Hastie and Tibshirani, 1990). GAMs where constructed in R using the gam function in the mgcv version 1.8.36 package (Wood, 2011) and drawn with ggplot2.

## 3. Results

### 3.1. Physico-chemical parameters

The pH level tended to be highest during the summer in Lake St. Clair and Lake Erie (Table 1), and reached a maximum value of 10 in the Western Basin during the summer of 2017. Temperature and DO were more variable across seasons than systems, and temperature was, on average, the highest in Lake Erie surface water during the summer. There was a gradient of decreasing Cond and nutrient concentrations along the water continuum. In particular, the Thames River had higher average TP compared to the rest of the sampled systems, especially in winter for the Upper Thames and in spring for the Lower Thames (Table 1). The highest TP concentration was measured in January 2018 at the most upstream site of the Thames River, reaching 519 μgL^−1^. TP decreased downstream in Lake St. Clair and averaged below 25 μgL^−1^ in Lake Erie, even in the Western Basin, which experiences annual cyanobacterial blooms and normally corresponds to a mesotrophic state. However, a brief peak of 79.3 μgL^−1^ TP was measured during spring 2017 in the Western Erie Basin. In fact, the average TP concentration in the Western Basin was only marginally higher than observed in the two other basins during the sampling period. A similar trend was observed for TDP, where concentrations were on average higher in the Thames River than in the rest of the aquatic continuum. No TN data were collected in the Thames River from 2016 to 2018, so reported values are only based on 2019 sampling. This showed a clear gradient of decreasing TN and TDN concentrations from the Thames River to Lake St. Clair and Lake Erie, where concentrations were on average one order of magnitude lower than in the Thames River (Table 1).

Chl*a*, which can be used as a proxy for algal biomass, was on average higher in the Thames River and Lake St. Clair during the spring, and decreased in summer and fall in all systems. Maximum levels of chl*a* were found in the Upper Thames River during the fall. In Lake Erie, chl*a* levels were on average always higher in the Western Basin compared to the Central and Eastern basin.

### 3.2. Microbial and cyanobacterial community structure and composition

The bio-informatics analysis recovered 67,847 ASVs present in 276 samples (85 in the Upper Thames, 55 in the Lower Thames, 62 in Lake St. Clair, 14 in Detroit River, and 60 in Lake Erie). Since there was a strong overlap among the centroids for the 4 years in dispersion plots (Supplementary Figure S1A), all further analyses were performed using the collated data for the 4 years.

**Figure 2:**
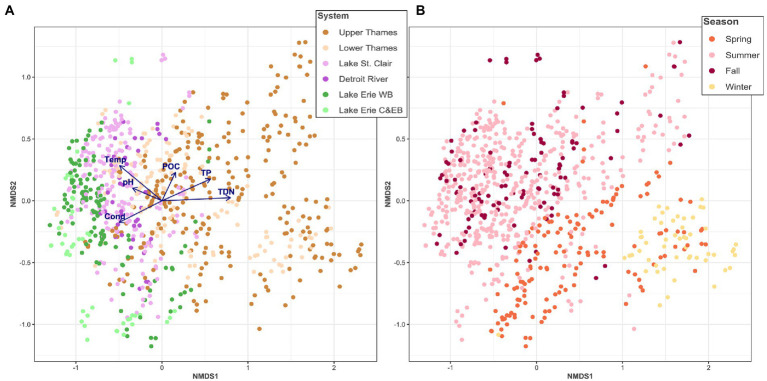
Non-metric multidimensional scaling (NMDS) of the microbial community based on Bray-Curtis distance, colored as a function of the system sampled overlaid with the significantly correlated environmental variables **(A)** and colored as a function of the season **(B)**. WB stands for Western Basin and C&EB for Central and Eastern Basin, respectively.

Microbial community composition was significantly different among the different systems (PERMANOVA *R*^2^ = 0.21, *p* < 0.01) and presented a longitudinal dissimilarity gradient along the hydrological continuum, as pointed out by two different multivariate ordinations (Figure 2A; Supplementary Figure S1B). Samples from the same system were more similar to each other with dissimilarity increasing from the Upper Thames River to Lake Erie’s Western Basin. The Central and Eastern Basins marginally clustered with the rest of the Lake Erie samples (Figure 2A). Cond, pH, Temp, POC, TP, and TDN were significantly correlated with the community spatial structuring (*p* < 0.01 for all variables). Our analyses indicated that the Thames River microbial community was influenced by higher nutrient concentrations, while those of Lake St. Clair and Lake Erie appeared to be mediated by higher pH, Cond, and temperature. Across the whole aquatic continuum, the microbial community showed distinct patterns across seasons (PERMANOVA R^2^ = 0.15, p < 0.01), although there seemed to be more overlapping across season than system, for example, between summer and fall (Figure 2B; Supplementary Figure S1C).

The microbial and cyanobacterial communities showed the same type of spatial patterns, as inferred by the Mantel correlations, which suggested that both were significantly correlated with spatial extent. Both communities showed the highest positive and significant spatial autocorrelation across samples that were less than 7 km apart (Table 2). Spatial autocorrelation stayed positive and mostly significant for samples within a geographic distance of less than 79 km, suggesting that within this distance, communities were more similar to each other than by chance. Beyond this ~79 km radius, the Mantel r became negative, indicating that beyond this distance, communities were more distinct from each other than by chance. Variance partitioning analysis was used to disentangle the influence of spatial vs. environmental variables. Environmental variables that were measured in every system (TDN, Cond, Temp, TDP, pH, TP) and the 12 PCNM axes representing the spatial variation were selected by the forward selection as significant explaining factors (*p* < 0.01 for each variable) for the variation partitioning analysis. This analysis explained 35% of the microbial community composition, and attributed 24% (*p* < 0.01) of the variance in microbial community to the environmental and 23% (*p* < 0.01) to the spatial variables, with a 12% intersection between the two. This indicates that 12% (*p* < 0.01) of the variation was solely due to the measured environmental variable and 11% (*p* < 0.01) solely to the geographic location. Sixty-five percent of the variation was left unexplained.

**Table 2:** Mantel *r* value for the whole microbial and cyanobacterial community as a function of the geographic distances with corresponding value of *p* <0.05 (*), < 0.01 (**) and < 0.001 (***).

Distance (km)	Mantel *r*	
	Microbial community	Cyanobacterial community
7	0.13***	0.14***
22	−0.001	−0.002
36	0.02*	0.02**
50	0.05**	0.06**
65	0.03**	0.03**
79	0.007	−0.0002
94	−0.03**	−0.03**
108	−0.08**	−0.09**
123	−0.05**	−0.01

**Figure 3:**
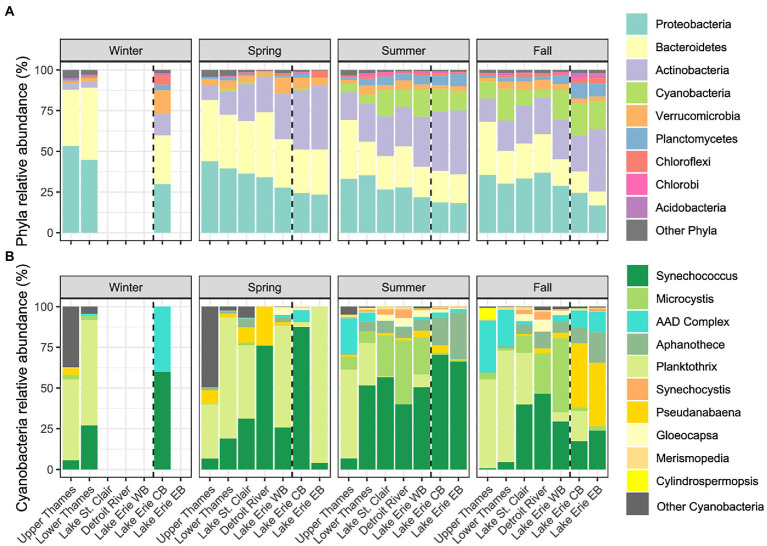
Relative abundances of the main microbial phyla expressed as % of the microbial community **(A)** and cyanobacterial genera expressed as % of the cyanobacterial community **(B)** along the water continuum and as a function of the different seasons. The Western Basin (WB) is separated by a dotted line from CB for the Central Basin (CB) and Eastern Basin (EB). Not all systems were sampled during winter (for sampling frequency see Supplementary Table 1).

The following proportions of phyla, genera, and ASVs represent average relative abundances of the 4 years of sampling with the standard deviation in parentheses. The phylum Proteobacteria dominated the Upper Thames River, representing 53% (9%) of the winter microbial community, and then decreasing in relative abundance through the water continuum as observed in each season (Figure 3A). A similar trend was observed for the Bacteriodetes, whereas the Actinobacteria and Cyanobacteria seemed to follow the opposite trend and increased in relative abundance from the Upper Thames River to Lake Erie. Cyanobacteria were further observed to be more abundant during summer and fall than in winter and spring. Here, the different cyanobacterial genera were classified using the BOLD database constructed with species representing a diverse array of cyanobacteria (Ivanova et al., 2019). Taxonomic assignment remained problematic for sequences corresponding to the genera *Anabaena*, *Aphanizomenon*, and *Dolichospermum*, which were then classified as the *Anabaena-Aphanizomenon-Dolichospermum* complex (AAD complex). During winter, the relative abundance of cyanobacteria was very low and only reached 0.3% of the microbial community in Lake Erie (only one winter sample available) and was mostly composed of *Synechococcus* and the AAD complex (60 and 40% of the cyanobacterial community, respectively; Figure 3B), while *Planktothrix* was dominant in the Upper and Lower Thames River (50% and 65% of the cyanobacterial community, respectively). In spring, the relative abundance of cyanobacteria reached a maximum of 1.4% (2%) in the Lower Thames River (Figure 3A), and was mostly composed of a mix between the genera *Planktothrix* and *Synechococcus* along the entire water continuum (Figure 3B). In summer, cyanobacterial relative abundance increased in the whole water continuum, reaching up to 17% (11%) in the Western Basin of Lake Erie (Figure 3A), and was mostly composed of the genera *Synechoccocus* and *Microcystis*, which increased in relative abundance from the Upper Thames River to the Western Basin of Lake Erie. At this season, *Synechoccocus* represented 57% of the cyanobacterial community in Lake St. Clair, 40% in the Detroit River, 50% in the Western Basin of Lake Erie, and 68% in Lake Erie Central and Eastern Basin, while *Microcystis* was the second most abundant genus with 25% of the cyanobacterial community in Lake St. Clair, 39% in the Detroit River, and 23% in Lake Erie Western Basin (Figure 3B). *Microcystis* was poorly represented in the central and eastern basin of Lake Erie, which were mostly dominated by *Synechococcus*. In contrast, *Planktothrix* was the most abundant cyanobacterial genus in the Upper Thames River, reaching 55% of the cyanobacterial community. During the fall, the cyanobacterial community stayed relatively abundant reaching 18% (14%) of the Lake Erie’s Western Basin, with *Microcystis* as the dominant cyanobacterial genus (*ca.* 45% of the cyanobacterial community), while *Pseudanabaena* dominated in the Central and Eastern Basins. *Planktothrix* was dominant in Lake St. Clair composing 32% of the cyanobacterial community.

### 3.3. Microbial connectivity within the watershed

The genetic connectivity was assessed amongst part of the system that were closely connected from upstream and downstream, and because of their poor connection with the rest of the water continuum, notably highlighted by the NMDS analysis (Figure 2A), Lake Erie’s Central and Eastern Basins were removed from subsequent analyses.

**Figure 4:**
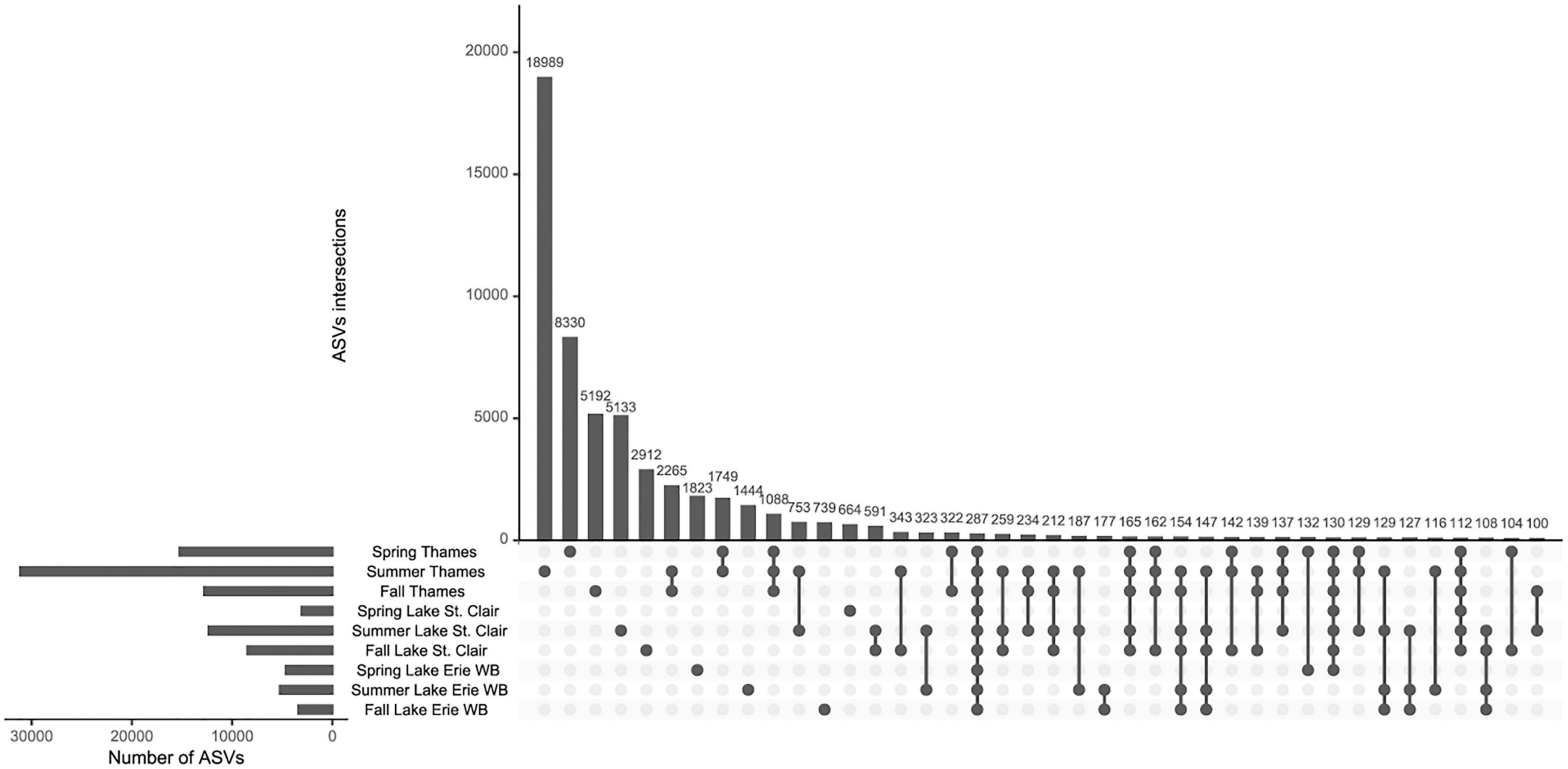
Intersection of ASVs across systems and seasons. Horizontal bars indicate the total number of ASVs for each system at each season and vertical bars represent the number of ASVs in the category designated by the dot below. Detroit River samples were lumped with Lake St. Clair samples and winter samples were removed due to lack of system represented. Only intersections of more than 100 ASVs are shown.

The highest number of ASVs was found in the Thames River, followed by Lake St. Clair (including the Detroit River) and Lake Erie (Western Basin; Figure 4). In each system, the number of ASVs was the highest during the summer, but overall, few were shared among systems and seasons. The data showed a ‘core microbiome’ of 287 ASVs shared across all systems and seasons, which represented less than 1% of all the 67,847 ASVs identified. The relative abundance of this ‘core microbiome’ varied from 10% of the community during the winter in the Upper Thames, to 77% during the spring in the Detroit River, and was composed of the same dominant phyla identified in Figure 3. Amongst those, the proportion of the cyanobacterial phyla in this core was from 0.04% of the microbial community during the winter in both Upper and Lower Thames, to 13% of the community during the fall in the Lower Thames. During spring and summer, the greatest number of ASVs were unique to the Thames River system and not found elsewhere along the water continuum. A smaller number of ASVs seemed to be specific to downstream Lake Erie Western Basin, and this number was the highest during spring (1823). The summer community in Lake Erie’s Western Basin had more ASVs in common with the summer community of Lake St. Clair and the Thames River or the fall community of Lake Erie than with the Western Basin of Lake Erie spring community.

**Figure 5:**
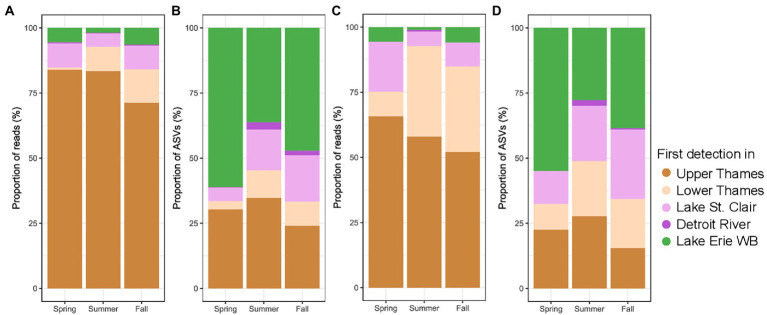
Proportion microbial reads **(A)** and ASVs **(B)**, as well as Cyanobacterial reads **(C)** and ASVs **(D)** in the Lake Erie Western basin at different season, classified as a function of the most upstream system they were first detected in (WB stands for Western Basin). Winter samples were removed due to lack of system represented.

The composition of the microbial community in Lake Erie’s Western Basin was highly connected to the rest of the watershed in terms of a high proportions of the reads (Figure 5A). A smaller proportion of ASVs (Figure 5B) in Lake Erie’s Western Basin were also detected in the upstream systems. The same pattern was observed for the cyanobacterial community (Figures 5C,D). In all seasons, the Thames River contributed the highest proportion of reads in the Western Basin of Lake Erie for both the whole microbial and the cyanobacterial community followed by Lake St. Clair and Lake Erie, while the contribution of the Detroit River was minimal. On the other hand, the greatest proportion of the bacterial and cyanobacterial ASV, at least in spring and fall, were first and only identified in the Lake Erie’s Western Basin.

**Figure 6:**
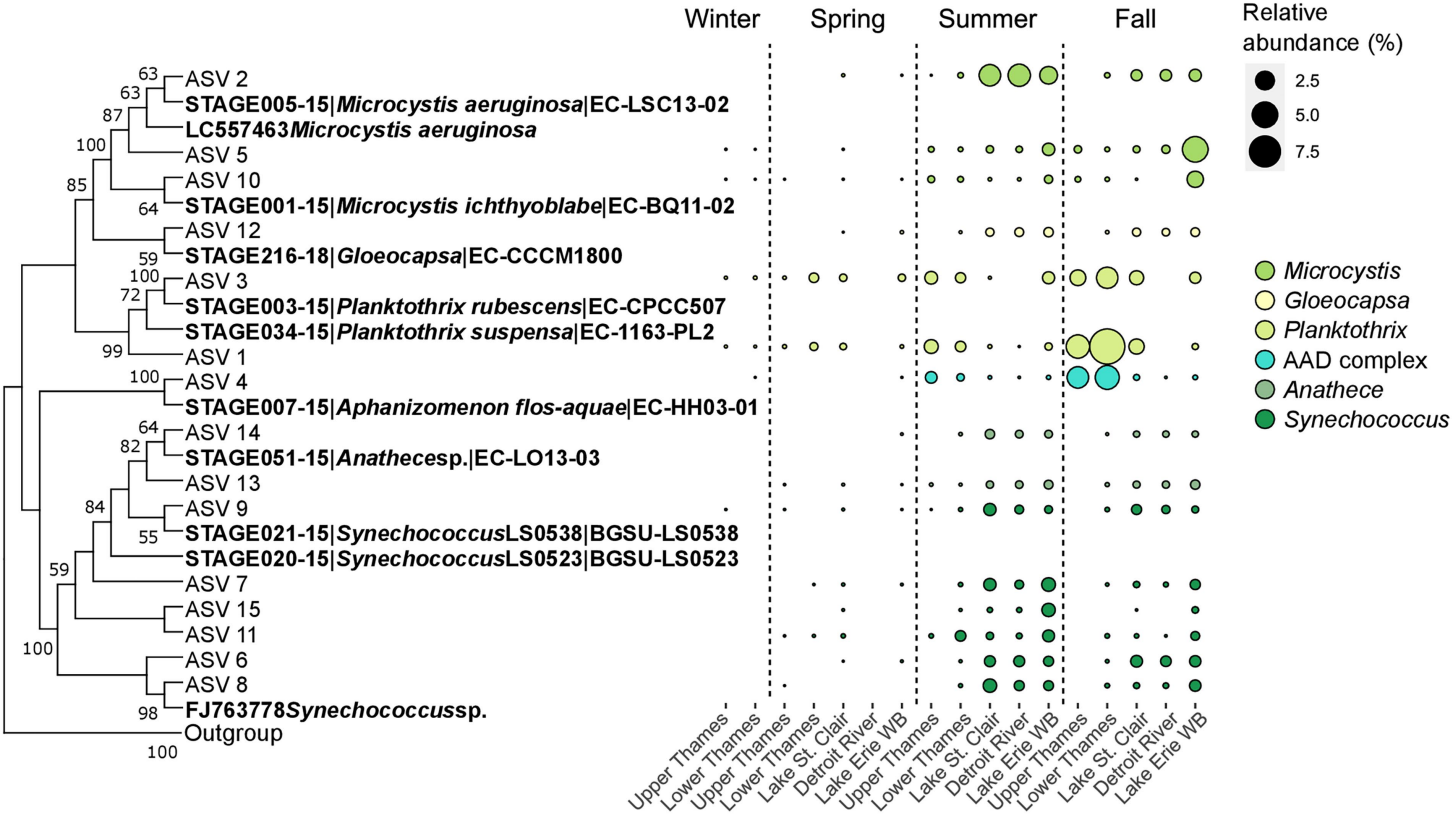
Neighbor-joining bootstrapped phylogenetic tree of the 15 most abundant cyanobacterial ASVs with sequences from culture representative of the BOLD reference database and sequences downloaded from GenBank with only bootstrap values above 50% from the 1,000 replicated trees indicated at the nodes of branches **(A)**. Corresponding relative abundance in % of the microbial community of the single ASVs along the water continuum and across season colored based on their genera **(B)**. WB stands for Western Basin.

Genetic connectivity within the watershed was further explored for the 15 most abundant cyanobacterial ASVs using a phylogenetic analysis. Those ASVs clustered with the genera *Microcystis*, *Gloeocapsa*, *Planktothrix*, *Aphanizomenon*, *Anathece* and *Synechococcus* (Figure 6A). Three ASVs (# 2, 5, and 10), clustering with representative of the genus *Microcystis*, seemed to dominate in different parts of the aquatic continuum and at different times (Figure 6B), and could generally be detected along the water continuum. During summer, ASV 2 started to become dominant in Lake St. Clair and reached 3.4% (3.8%) of the microbial community, followed by 3.7% (3.2%) in the Detroit River and 2.2% (2.5%) in Lake Erie’s Western Basin, it only accounted for less than 0.01% of relative abundance in the Upper Thames River. On the other hand, the other two ASVs clustering with *Microcystis* (ASV 5 and 10) seemed to become more abundant in Lake Erie during the fall, reaching 5% (8.3%) and 1.8% (1.4%) of the microbial community, respectively, but stayed in lower relative abundance in Lake St. Clair. ASV 12 clustered with the culture representative^3^ identified as *Gloeocapsa*, which peaked in relative abundance in the Western Basin during summer but represented only 0.5% (0.7%) of the microbial community. The next cluster in the phylogenetic tree contained two ASVs of the genus *Planktothrix* (ASV 1 and 3) that both dominated in the Thames River during summer and fall, especially ASV 1 which accounted for 9.9% (10.3%) of the microbial community in the Lower Thames River during fall. ASV 4 clustered closely with the sequence of the culture representative *Aphanizomenon flos-aquae* that was more abundant in the Thames River, reaching 4.4% (7.6%) of relative abundance in the Lower Thames during the fall. This ASV was also present in Lake St. Clair and Erie, although in lower relative abundance. Finally, the last cluster of the phylogenetic tree gathered sequences of 6 different ASVs clustering with the genus *Synechococcus* and two with the genus *Anathece*, both genera belonging to the Order Synechococcales. Those ASVs started to become more abundant in Lake St. Clair, followed by the Detroit River, and Lake Erie during summer and fall, and some were not detectable at all in the Upper Thames River (ASVs 7, 15 and 6).

### 3.4. Influence of environmental variables on cyanobacterial dynamic

Because of the non-linear nature of the relationships, Generalized Additive Models (GAMs) were used to test those correlations, and, although all the relationships presented here were significant (*p* < 0.01), the explanatory power of the *R*^2^ remained relatively low (from 0.02 to 0.47), and no clear pattern was observed between the set of selected environmental variables and the relative abundance of dominant Cyanobacteria (*Microcystis*, *Synechococcus*, and *Planktothrix*). Compared to *Planktothrix*, both *Microcystis* and *Synechoccocus* genera seemed to peak at lower nutrient (N and P) concentrations which corresponded to concentrations found in Lake Erie in comparison to the Thames River (Supplementary Figure S2). It was noteworthy that across all sites, the relative abundance of both *Microcystis* and *Synechoccocus* tended to decrease as nutrient concentrations increased. Although significant and positively correlated, temperature had an extremely low *R*^2^ for *Microcystis* (*R*^2^ = 0.02) compared to *Synechococcus* (*R*^2^ = 0.3). No clear relationship was observed between cyanobacterial relative abundance and pH. Finally, the relative abundance of *Microcystis* seemed to peak at a TN/TP mass ratio ≥ 30, which generally corresponds to the multi-annual average TN/TP mass ratio (35) of Lake Erie’s Western Basin, while the other systems had higher average TN/TP mass ratio values (Central and Eastern Basin of Lake Erie = 47, Lake St. Clair = 40, Detroit River = 41, Lower Thames = 48, and Upper Thames = 80). However, no clear trend was observed between *Synechoccocus* or *Planktothrix* relative abundances and nutrient mass ratios.

## 4. Discussion

Here we showed how geographic distance and seasonal variations, as well as environmental variables, are shaping the microbial communities in the Thames River–Lake St. Clair-Lake Erie continuum, and found a high degree of connectivity between upstream and downstream systems, despite the extended geographic distance. We found support for our hypothesis that upstream watershed can be a source of recruitment for Lake Erie’s microbial and cyanobacterial communities, though stronger for microbial than cyanobacterial communities, and not excluding other tributaries or sediments as more proximal sources of microbial communities. Overall, our results provide support for the importance of watersheds (and associated land use activities) as a major influence for Lake Erie’s communities.

The Thames River–Lake Erie continuum represents a strong gradient of environmental conditions reflecting change in land use and urbanization. Amongst Canadian tributaries to Lake St. Clair and western Lake Erie, the Thames River contributes greatest to P loading consistent with elevated discharge draining a watershed dominated by row crop agriculture (Van Rossum and Norouzi, 2021; Kao et al., 2022). Amongst all tributaries discharging into Lake Erie, the Thames River ranks 3^rd^ behind the Maumee and Sandusky Rivers in contributing TP and SRP to Lake Erie (Maccoux et al., 2016; Figure 1). Although the largest annual hydrological loads to Lake St. Clair come from the St. Clair River (average 2016–2019 discharge of 6,122 m^3^/s), which flows directly from Lake Huron, the Thames River has a disproportionate effect on the TP load, to the extent that P reduction in the Thames River basin will be more effective in reducing the P export from Lake St. Clair than from the equivalent in-flow Sydenham or Clinton Rivers (Bocaniov et al., 2019). Lake St. Clair has a water residence time of ~9 days, and due to it shallow depth, is subjected to frequent vertical mixing, yet nutrient loads from its tributaries are not homogeneously mixed and vary seasonally and spatially (Bocaniov et al., 2019). Lake St. Clair has also been shown to be a sink for nutrients retaining up to 20% of the TP load (Bocaniov et al., 2019; Scavia et al., 2019), but altogether with loads from Canada and the US still contributes up to 41% of the annual TP load to Lake Erie’s Western Basin *via* the Detroit River (average 2016–2019 discharge of 6,383 m^3^/s), which is the second in importance after the Maumee River which contributes up to 48% (Scavia et al., 2016).

### 4.1. Microbial and cyanobacterial community structure and composition

We observed longitudinal and seasonal changes in the aquatic microbiome within the Lake Erie watershed from the Upper Thames River to Lake St. Clair and Lake Erie. We observed a low relative abundance of cyanobacteria in winter and spring, followed by an increase in their relative abundance during summer and fall which is similar to observations elsewhere when cyanobacterial blooms tend to occur due to higher temperature optima and preference for strong stratification (Paerl and Huisman, 2009). Nevertheless, PERMANOVA analyses suggested that the spatial pattern was more influential than the seasonal pattern on the microbial community structure. While part of the same water continuum, the aquatic systems sampled here represents a gradient of environmental conditions and trophic status, with the Thames River draining nutrients from its predominantly agricultural watershed, which are gradually diluted downstream in Lake St. Clair and Erie. Lake Erie itself represents a longitudinal gradient in trophic status where its Western Basin is mesoeutrophic while the Central and Eastern Basins trend to oligomesotrophic (Dove and Chapra, 2015). The spatial directional pattern observed for the microbial community composition has already been observed in previous meta-community studies on boreal inland waters (Ruiz-Gonzalez et al., 2015), Arctic systems (Crump et al., 2012; Comte et al., 2018), and across the Great Lakes (Rozmarynowycz et al., 2019; Paver et al., 2020). Those studies, together with our findings on the most important tributary on the Canadian side, highlighted the importance of hydrological processes in shaping the microbial community structure in aquatic continuums.

The mantel correlations showed that, for both microbial and cyanobacterial communities, the highest spatial autocorrelation occurred within a radius of 7 km (Table 2). At a wider spatial scale, within a 79 km radius, communities remained more similar than by chance, indicating a certain degree of selection of the microbial community within similar types of system. This radius matches the limit of each system sampled (Upper and Lower Thames, Lake St. Clair, Detroit River and Lake Erie’s Western Basin), where the greatest distance between two samples of a similar system was 54 km between the two most distant Lower Thames samples. Beyond 79 km, a distance-decay pattern started to appear, which indicate dispersal limitation and system filtering as observed in other aquatic ecosystems (Logares et al., 2018). Even within Lake Erie, where despite having the shortest residence time of all the Great Lakes (ca 2.7 years; Quinn, 1992), there was a distinction in the microbial and cyanobacterial community structure between the Western Basin and the rest of the lake, probably driven by the difference in trophic status and hydrology. Collectively, the results indicated that both microbial and cyanobacterial community structures were significantly correlated with the spatial distances and that, beyond a threshold of 79 km which also corresponded to samples within the same system, communities switched from being more similar to more dissimilar than by chance.

Environmental variables including conductivity, temperature, pH, particulate organic matter and nutrients (TP and TDN) have been identified as variables which significantly influenced microbial community structure. Water chemistry parameters often shape aquatic system due to their direct impact on microbial metabolism. Nutrients, and by extension, trophic status, have a prime effect on microbial community structure (Lindström, 2000) and diversity, with higher richness associated with more eutrophic lakes (Kiersztyn et al., 2019), yet richness tends to decrease with N fertilization (Schulhof et al., 2020). As an integrator of landscape properties, pH is often identified as one of the main factors influencing microbial community structure (Lindström et al., 2005; Fierer and Jackson, 2006; Logue and Lindström, 2008; Niño-García et al., 2016). However, variance partitioning analysis of our data indicated that environmental variables *per se* only explained 12% of the variation observed within the microbial community, and this proportion reached 35% when accounting for both environmental and spatial patterns. This relatively low explanatory power is not uncommon when investigating factors that influence aquatic microbiomes (Marmen et al., 2020), and highlights the importance of examining other factors that can play key roles in microbial community assembly, for example, by including a more complete characterization of the hydrological properties of the systems, including the lag time (Lindström et al., 2005; Marmen et al., 2020), and taking into account the biotic interactions with co-occurring microbes or with higher and lower trophic levels (Louca et al., 2016).

The rest of the aquatic microbiome community interacts extensively with cyanobacteria, notably through mutualistic interactions (Eiler and Bertilsson, 2004; Woodhouse et al., 2016; Berg et al., 2018), toxin degradation (Mou et al., 2013), or predation (Rashidan and Bird, 2001); and bloom events may create a disturbance to this microbiome (Berry et al., 2017). The typical dominant freshwater bacterial phyla, i.e., Proteobacteria, Bacteroidetes, Actinobacteria, and Verrucomicrobia, are often found associated with cyanobacterial blooms (Eiler and Bertilsson, 2004; Bagatini et al., 2014; Tromas et al., 2017; Li et al., 2020), while Proteobacteria have been associated with two strains of cultured cyanobacteria (*Microcystis* and *Cylindrospermopsis*; Bagatini et al., 2014). However, in our case, Proteobacteria’s relative abundance was weakly and negatively correlated with cyanobacteria’s (SparCC *ρ* = −0.05, *p* < 0.01), and Proteobacteria were found in higher relative abundance in the Thames River compared to the downstream systems. Zhu et al. (2019) also noticed a significant negative correlation between the two phyla relative abundances, suggesting a potential competitive interaction. Negative correlations have been observed between cyanobacteria and Actinobacteria’s relative abundances (Ghai et al., 2014; Matson et al., 2020), with Actinobacteria occurrence being associated with less eutrophic systems (Haukka et al., 2006), although actinobacterial clades were abundant in eutrophic Lake Taihu (Tang et al., 2017). Yet, some taxa affiliated with the Actinobacteria phylum co-occur with cyanobacterial blooms (Woodhouse et al., 2016) or having the relative abundance of certain clade strongly correlated, either positively or negatively, to bloom occurrence (Berry et al., 2017). In our case, Actinobacteria’s relative abundance was positively correlated with cyanobacteria’s (SparCC *ρ* = 0.4, *p* < 0.01) and were found in higher relative abundances in the downstream Lakes St. Clair and Erie. Some taxa belonging to the Verrucomicrobia have been shown to display a higher diversity in the presence of cyanobacteria (Parveen et al., 2013) and are known to degrade algal polysaccharides and organic matter (Bagatini et al., 2014; Woodhouse et al., 2016). However, in our dataset, there was no significant relationship between relative abundances of Verrucomicrobia and Cyanobacteria. Bacteroidetes have also been shown to increase in absolute and relative abundance during or after a cyanobacterial bloom, especially the Sphingobacteria and Flavobacteria classes (Newton et al., 2011; Bagatini et al., 2014). However, we observed a weak but negative correlation between Bacteroidetes and Cyanobacteria’s relative abundances (SparCC *ρ* = −0.07, *p* < 0.01), which suggests that other factors not investigated in this study are important. In contrast, the relative abundance of Planctomycetes and Cyanobacteria were positively correlated (SparCC *ρ* = 0.5, *p* < 0.01), consistent with other reported associations between Planctomycetes and phytoplankton blooms (Eiler and Bertilsson, 2004). Because of their involvement in nutrient cycling, the aquatic microbiome is likely to play a crucial role in bloom occurrence and duration, and it has already been highlighted that the rest of the aquatic microbiome could be a better predictor of cHAB events than environmental factors (Tromas et al., 2017). More studies are needed to explore those interactions in further detail, notably by including eukaryotic microbes, and unveiling the mechanisms underlying these interactions.

Microscopy and molecular tools have been found to identify the same predominant taxa of cyanobacteria, with the exception of pico-cyanobacteria which are underestimated by microscopy (MacKeigan et al., 2022). Here, molecular tools allowed detection of *Synechococcus* as one of the dominant genera of cyanobacteria in Lake Erie’s watershed, along with *Microcystis*, *Planktothrix*, and *Aphanizomenon*, which were already identified in Lake Erie using microscopy (Millie et al., 2009; Chaffin et al., 2011; Allinger and Reavie, 2013; Davis et al., 2015). Other studies based on meta-barcoding results also identified *Microcystis*, *Planktothrix*, *Synechococcus*, *Aphanizomenon*, and *Dolichospermum* as dominant genera of cyanobacteria in river mouths (Sandusky and Maumee) and in the Western and Central Basins of Lake Erie (Berry et al., 2017; Salk et al., 2018; Jankowiak et al., 2019; Rozmarynowycz et al., 2019; Matson et al., 2020). *Synechoccocus* and *Microcystis* were found to co-occur as dominant genera in Lake St. Clair, Detroit River, and Lake Erie (Figure 3B), and this co-occurrence was already observed in Lake Erie (Berry et al., 2017; Rozmarynowycz et al., 2019) and in Lake Taihu (Ye et al., 2011). *Synechococcus* also initiated the 2014 cyanobacterial bloom in the Western Basin of Lake Erie and stayed abundant until the end of the bloom (Berry et al., 2017). In the Thames River, sequences corresponding to the genera *Planktothrix* and *Aphanizomenon* were found in high relative abundances (Figure 6B). To date, very few blooms have been reported in the Thames River, although there is increasing evidence that the river experiences recurrent blooms (Crevecoeur, Molina and Watson, pers. communication, Supplementary Figure S3). A bloom reported in the lower portion of the river around Chatham and identified by microscopy was dominated by *Aphanizomenon flos aquae* and *Planktothrix agardhii* (McKay et al., 2020), which is consistent at the genus level with what we found in our genomic data from 2016 to 2019. However, our data also suggest the presence of ASVs clustering with *Planktothrix rubescens* and *P. suspensa* (Figure 6A). As a comparison, *Plantkothrix* has also been reported as abundant on the US side of Lake Erie, specifically in the Sandusky Bay (Davis et al., 2015; Salk et al., 2018; Jankowiak et al., 2019). These observations suggest that *Microcystis* and *Synechococcus* might be well adapted to lake conditions and *Planktothrix* to more turbid river-type conditions. Our data also highlight the existence of complex assemblages of different strains of cyanobacteria that successively dominate the water continuum through space and time. Further investigation is needed to determine if these different strains and ASVs respond in the same way to environment triggers for toxin production.

### 4.2. Microbial connectivity within the watershed

While the connectivity between Lake Erie and its tributaries has been clearly identified in terms of P loading (Maccoux et al., 2016), the connectivity of the aquatic microbiome and the contribution of riverine Bacteria to the lake microbiome remain uncertain. The Thames River holds a high proportion of microbial taxa that were only identified in the upstream river but not in any of the downstream systems (Figure 4). Lake St. Clair appeared to act as a filter and is also influenced by other sources not taken into account here, such as the upstream Huron corridor. In summer and fall, there were more taxa shared between Lake Erie and the rest of the watershed than between Lake Erie in the spring. This highlights the seasonal succession and change in aquatic microbial community.

Within a watershed, the most commonly observed patterns of microbial community assembly are mass effect, i.e., massive advection of numerically dominant microbes by passive transport, and species sorting (Lindström and Bergström, 2004; Zhao et al., 2021). In our case, the majority of microbial reads found in the Western Basin of Lake Erie were likewise identified from the Thames River (Figure 5). This observation allows to unveil the impact of dispersal within the watershed in general, but this does not rule out other sources, notably amongst other more proximal tributaries, or with higher discharge, and/or the sediment, as ASVs can be shared across multiple watersheds (Urycki et al., 2022). Other studies have classified reads and ASVs as a function of the system in which they were first detected (Crump et al., 2012; Ruiz-Gonzalez et al., 2015; Matson et al., 2020), and our data clearly indicated that mass effect played an important role in the downstream microbial community assemblage. This phenomenon is prevalent when dispersion is high due to elevated levels of connectivity between the upstream river community and the downstream lake (Adams et al., 2014; Ruiz-Gonzalez et al., 2015). However, in lake systems, where water flow generally decreases and residence time increases, species sorting occurs (Zhao et al., 2021). Indeed, our data showed that certain cyanobacterial ASVs, mainly ASVs 2, 5 and 10, clustering with *Microcystis* isolates, were found in low relative abundance in the Upper Thames River, but became dominant in the Western Basin of Lake Erie (Figure 6), suggesting the lake conditions were selecting these cyanobacterial ASVs to become dominant. This genetic connectivity between upstream and downstream has already been highlighted for microcystin producers such as *Microcystis*, *Planktothrix*, and *Anabaena* in Lake Erie, Lake St. Clair, and Lake Ontario based on the *mcyA* gene responsible for microcystin production (Davis et al., 2014). Our data suggest that this connectivity extends beyond the Great Lakes to the upstream tributaries, and also across different seasons. As to whether Lake Erie *Microcystis*-dominated harmful blooms are seeded from the river, based on our data it seems unlikely that the Thames River is the sole source for those *Microcystis* ASVs given the extremely low relative abundance in which they were recovered. Additional sources likely contribute to the rapid development of summer and fall blooms in the Western Basin of Lake Erie, for example, recruitment from the sediment (Kitchens et al., 2018), or other tributaries (Maumee, Sandusky, and St. Clair Rivers), which still need to be investigated as both evidences supporting (Bridgeman et al., 2012) or refuting (Kutovaya et al., 2012) riverine seeding from the Maumee River have been documented.

### 4.3. Influence of environmental and spatial patterns

In our study, no clear pattern was observed between cyanobacteria relative abundance and nutrient concentrations, although our results suggested that *Planktothrix* relative abundance peaked at higher N and P concentrations than *Microcystis* and *Synechococcus* (Supplementary Figure S2). *Planktothrix* also dominated in an experiment with nutrients (N, P) addition while, under the same conditions, *Microcystis* decreased in relative abundance (Jankowiak et al., 2019). A similar observation was made by Harke et al. (2016) based on shipboard incubations of samples collected from a bloom site on Lake Erie’s Western Basin. These authors found that *Microcystis* abundance significantly decreased with P enrichment, while *Planktothrix* and *Dolichospermum* (formerly *Anabaena*) dominated samples collected from the high P environment near the mouth of the Maumee River. On the other hand, *Planktothrix* dominance has also been observed in the Sandusky river plume, where it’s capacity to accumulate N through cyanophycin compounds provided a competitive advantage under N limiting conditions (Hampel et al., 2019).

*Microcystis* seems to be more efficient at scavenging P, giving it an advantage to dominate at lower nutrient concentrations (Gobler et al., 2016; Harke et al., 2016), but it is also more adapted to stable water conditions because of its ability to regulate its buoyancy (Dokulil and Teubner, 2000). In contrast, *Synechococcus* is generally dominant in oligotrophic lakes (Ruber et al., 2016). It has been argued that the ability of some cyanobacteria to fix N provides a competitive edge in eutrophic systems with low N:P ratios (Smith, 1983), however this tenet has been debated (Downing et al., 2001). Our data showed generally negative correlations between N concentrations and the relative abundance of some non-diazotroph genera like *Microcystis* and *Synechococcus*, which showed higher relative abundances at lower N:P ratios (Supplementary Figure S2). These genera could be complemented by the presence of diazotrophic cyanobacteria and Proteobacteria (Davis et al., 2015; Li et al., 2020). Therefore, the presence, for instance, of N2-fixing *Aphanizomenon* and *Cylindrospermopsis* along the water continuum, could play a role in promoting the growth of *Microcystis* and *Synechococcus*. More investigations are needed to address those questions but molecular tools have the potential to provide more insight into the role of the entire microbiome, often overlooked in early studies.

Increasing temperature and longer warmer periods likely to happen due to climate change will also have an effect on cHABS, because a more stable thermocline and reduction in epilimnion mixing can lead to better light competition outcomes for cyanobacteria due to their ability to adjust their buoyancy. For instance, when nutrient concentrations are high enough to sustain bloom formation, climatic variables may become crucial for predicting bloom onset and duration, as was observed by Zhang et al. (2012), who found that increasing temperature, hours of sunshine, and reduction of wind speed in Lake Taihu lead to earlier onset and longer bloom duration. However, Taihu is a subtropical lake, and because Lake Erie has a temperate climate, the variables influencing its microbiome might be very different. Since our study was conducted across different seasons, we were likely to identify an effect of temperature. The relationship was positive and significant for *Microcystis*, *Synechoccocus*, and *Plantkothrix*, but the R^2^ of the GAMs were very low for *Microcystis* and *Planktothrix* (0.02 and 0.11, respectively); hence it is difficult to draw conclusions regarding the link between cyanobacteria relative abundance and temperature. However, climate projections predict that Lake Erie will have longer and more stable periods of stratification, which are likely to lead to increasing frequency and worsening impact of cHABs (Paerl and Huisman, 2008).

## 5. Conclusion

Here we used an integrated approach at the watershed scale to better understand the biological and ecological context shaping Lake Erie’s microbiome, with a special focus on the cyanobacterial community given Lake Erie’s long history of harmful algal blooms. This study showed how spatial and temporal variations, as well as environmental variables, are shaping the microbial communities in the Thames River–Lake St. Clair-Lake Erie continuum. In terms of community composition, gradual changes along the water continuum were observed for the whole microbial community. On a finer taxonomical level, distinct communities of cyanobacteria developed in the Thames River, mostly dominated by *Planktothrix*, compared to Lake St. Clair and Lake Erie, which were dominated by *Microcystis* and *Synechococcus*, notably during the summer. The high proportion of microbial reads shared between the Thames River and the Western Basin of Lake Erie suggested a high degree of connectivity and dispersal within the watershed in general, which probably extend to other tributaries, and where mass effect seemed to be an important driver of microbial community assembly. Nevertheless, distance-decay patterns of the Mantel correlations indicated some selection pressure within similar systems as it was notably observed for some cyanobacterial ASVs related to *Microcystis*. Despite being found in low relative abundance in the upstream river, *Microcystis* ASVs were selected by the conditions in the Western basin of Lake Erie, where they became progressively dominant. Genetic characterization of the community also showed that the dominant genera of cyanobacteria in Lake Erie watershed (*Microcystis*, *Synechococcus*, *Planktothrix*) were in fact composed of an assemblage of different ASVs becoming dominant at different times. These data further suggested that the two most abundant cyanobacterial genera in Lake St. Clair and Lake Erie, *Microcystis* and *Synechococcus*, stayed relatively abundant even at lower N and P concentrations than *Planktothrix*, which dominated in the higher nutrient regimes of the Thames River. Overall, there was still a large proportion of the variation observed in the microbial community structure that was not explained by spatial and environmental factors, demonstrating the importance of unexplored drivers of microbial and cyanobacterial community structure such as biotic interactions and hydrodynamics. The high connectivity and level of dispersion between the upstream, riverine and Lake Erie microbial and cyanobacterial communities highlight the need for watershed scale management to improve the water quality conditions in Lake St. Clair and the Western Basin of Lake Erie.

## 6. Data availability statement

The datasets presented in this study can be found in online repositories. The names of the repository/repositories and accession number(s) can be found at: https://www.ncbi.nlm.nih.gov/genbank/, PRJNA877648.

## Author contributions

SC, TE, SW, and JC designed study. SC, TE, LW, JC, ND, AD, KD, TF, RM, AZ, and JC collected data. SC and JC analyzed sequencing data, performed statistical analyses, and conceived of the manuscript. SC, TE, LW, SW, CG, and JC supervised the project and performed research. SC took the lead in writing the manuscript with help in editing and correcting from all the other authors. All authors contributed to the article and approved the submitted version.

## Funding

This study was funded under the Canada’s Genomics Research and Development Initiative (GRDI) & Great Lake Protection Initiative (GLPI), and also supported by grants from the Natural Sciences and Engineering Research Council of Canada (NSERC) (RGPIN-2019-03943; RGPIN-2020-06874), the Great Lakes Center for Fresh Waters and Human Health supported by NIEHS (1P01ES02328939–01), and NSF (OCE-1840715).

## Conflict of interest

The authors declare that the research was conducted in the absence of any commercial or financial relationships that could be construed as a potential conflict of interest.

## Publisher’s note

All claims expressed in this article are solely those of the authors and do not necessarily represent those of their affiliated organizations, or those of the publisher, the editors and the reviewers. Any product that may be evaluated in this article, or claim that may be made by its manufacturer, is not guaranteed or endorsed by the publisher.

## Footnotes

^1^https://wateroffice.ec.gc.ca/

^2^https://waterdata.usgs.gov/nwis

^3^http://www.boldsystems.org/
