# Supplementary material for: Spatio-temporal connectivity of the aquatic microbiome associated with cyanobacterial blooms along a Great Lake riverine-lacustrine continuum
Source: Front Microbiol. 2023 Feb 9;14:1073753. doi: 10.3389/fmicb.2023.1073753 (PMC9947797; doi:10.3389/fmicb.2023.1073753)
Supplement: Supplementary file 1 [file Data_Sheet_1.docx]

# Supplementary Table

**Supplementary Table 1.** Temporal distribution of sampling.

| **Year** | **Sampling campaign** | **Number of samples** | | | | |
| --- | --- | --- | --- | --- | --- | --- |
|  |  | Upper Thames | Lower Thames | Lake St. Clair | Detroit River | Lake Erie |
| 2019 | Apr. 30 -May 2 |  |  |  |  | 4 |
|  | May 29 - June 4 | 5 | 3 | 4 |  | 1 |
|  | June 23-27 | 5 | 3 | 4 | 1 | 1 |
|  | July. 8-10 | 5 | 3 | 1 |  |  |
|  | July 18-25 |  |  | 3 | 1 | 1 |
|  | Aug. 7 | 5 |  |  |  |  |
|  | Aug. 18-28 |  | 3 | 3 | 1 | 3 |
|  | Sep. 16-19 | 5 | 3 | 4 | 1 | 1 |
|  | Oct. 8-15 | 5 | 3 |  |  | 4 |
|  | 31-Oct. |  |  | 2 |  |  |
| 2018 | Jan.23 | 4 | 2 |  |  |  |
|  | Feb. 28 | 4 | 2 |  |  |  |
|  | Apr. 17 | 4 | 2 |  |  |  |
|  | May 29-31 | 4 | 2 | 4 | 1 | 1 |
|  | June 26-28 |  |  | 4 | 1 | 1 |
|  | July 17-18 | 4 |  | 5 |  | 1 |
|  | Aug. 14 | 5 | 2 |  |  |  |
|  | Aug 28-30 | 5 | 2 | 4 | 1 | 6 |
|  | Sep. 11-13 |  |  | 4 | 1 | 1 |
|  | Sep. 19-26 | 4 | 2 |  |  | 3 |
|  | Oct 10-11 |  |  | 4 | 1 | 1 |
| 2017 | Jan. 23 | 4 | 2 |  |  |  |
|  | May 2-12 | 5 | 2 |  |  | 5 |
|  | May. 30-31 |  |  | 4 | 1 |  |
|  | July 25-26 | 5 | 2 | 4 | 1 | 1 |
|  | Aug. 22-31 | 5 | 2 | 4 | 1 | 6 |
|  | Sep. 5-6 | 5 | 2 | 4 | 1 | 1 |
|  | Oct. 02-14 |  |  | 4 | 1 | 5 |
| 2016 | Feb. 16-18 |  |  |  |  | 3 |
|  | May 10-13 |  |  |  |  | 3 |
|  | July 11-13 |  |  |  |  | 3 |
|  | Aug. 17 | 5 | 3 | 4 |  |  |
|  | Aug. 30- Sep. 5 |  | 1 |  |  | 5 |
|  | Oct. 3 |  | 1 |  |  |  |

# Supplementary Figures

#
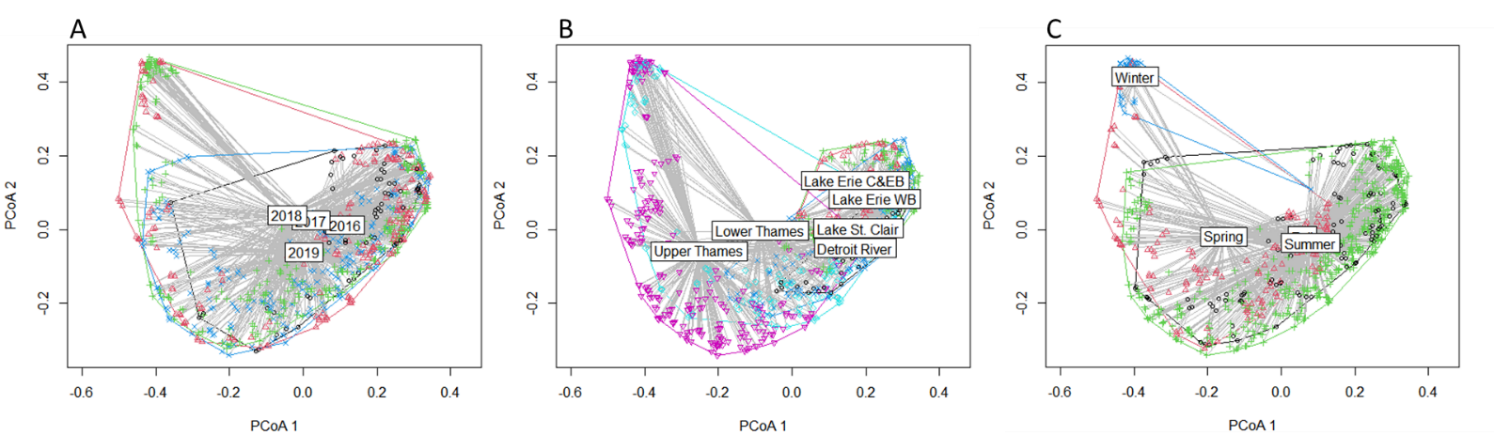


**Supplementary Figure 1.** Multivariate dispersion of samples according to the different years (A), system (B) and season (C) of sampling.


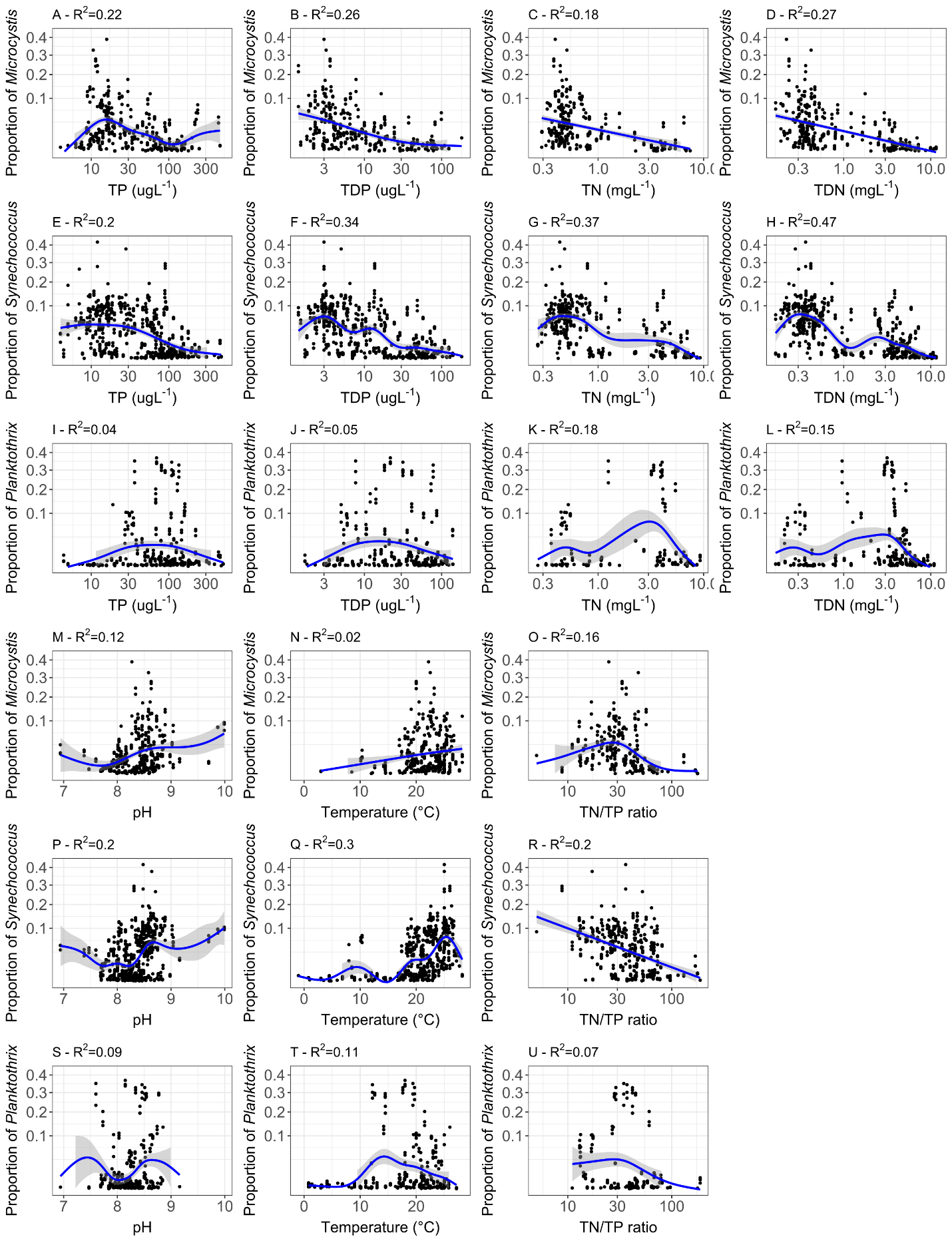


**Supplementary Figure 2.** Relationship identified with generalized additive models (GAMs) between relative abundance of *Microcystis* and TP (A), TDP (B), TN (C), TDN (D); *Synechoccocus* and TP (E), TDP (F), TN (G), TDN (H); *Planktothrix* and TP (I), TDP (J), TN (K), TDN (L); *Microcystis* and pH (M), Temperature (N), TN/TP ratio (O); *Synechoccocus* and pH (P), Temperature (G), TN/TP ratio (R); *Planktothrix* and pH (S), Temperature (T), TN/TP ratio (U). Shaded areas mean 95% confidence intervals. All relationships were statistically significant (p<0.01).


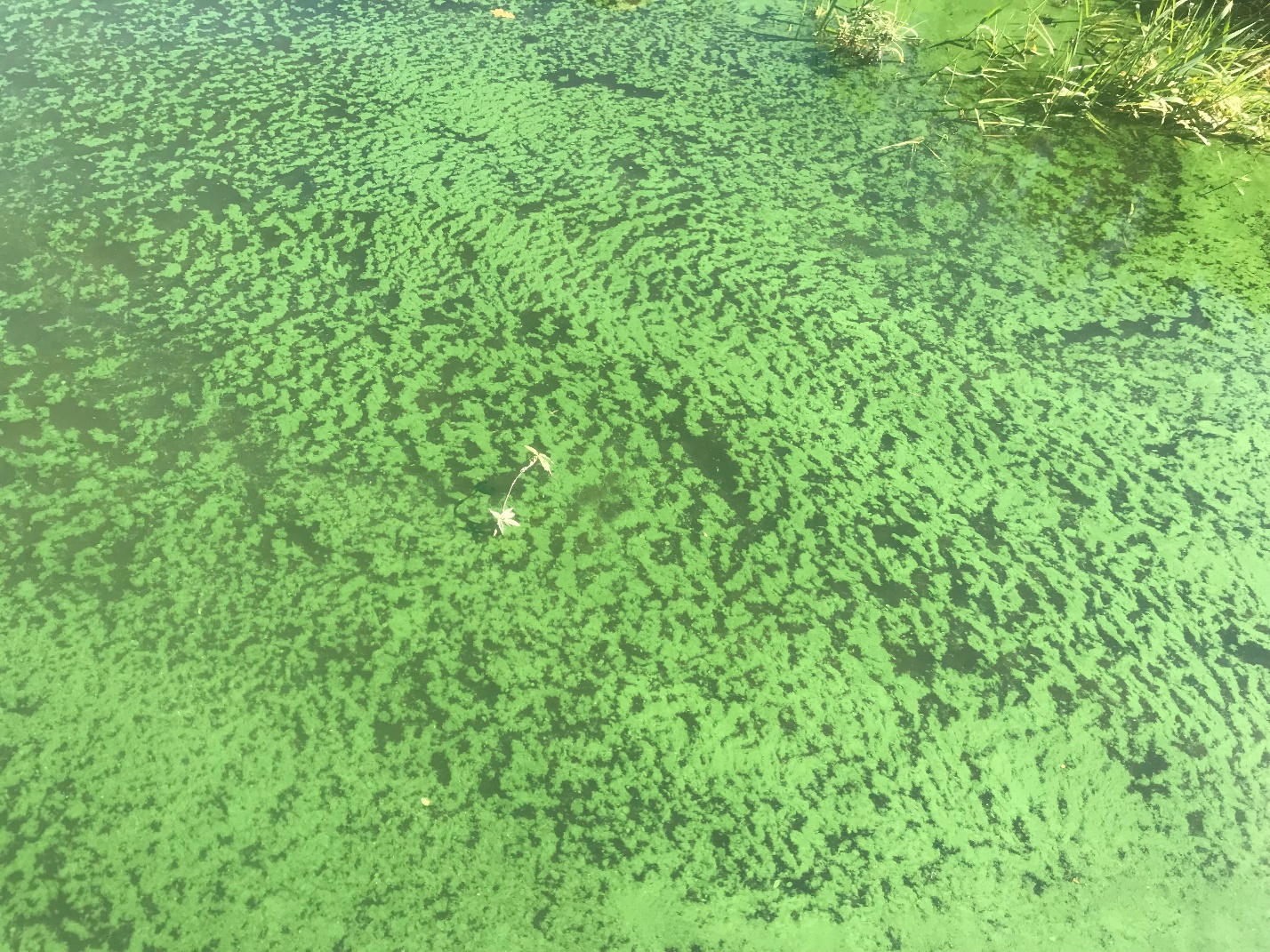


**Supplementary Figure 3.** Bloom observed in Fanshawe reservoir during the fall of 2019. Photo: Maria Molina.
